# In Vitro Potential of Clary Sage and Coriander Essential Oils as Crop Protection and Post-Harvest Decay Control Products

**DOI:** 10.3390/foods11030312

**Published:** 2022-01-24

**Authors:** Robin Raveau, Joël Fontaine, Abir Soltani, Jouda Mediouni Ben Jemâa, Frédéric Laruelle, Anissa Lounès-Hadj Sahraoui

**Affiliations:** 1 Unit of Environmental Chemistry and Interaction on the Living (UCEIV), University of the Littoral Opal Coast (ULCO), UR 4492, SFR Condorcet FR CNRS 3417, 50 Rue Ferdinand Buisson, 62228 Calais, France; robin.raveau@univ-littoral.fr (R.R.); joel.fontaine@univ-littoral.fr (J.F.); frederic.laruelle@univ-littoral.fr (F.L.); 2 Laboratory of Biotechnology Applied to Agriculture, National Agricultural Research Institute of Tunisia (INRAT), University of Carthage, Rue Hedi Karray, El Menzah, Tunis 1004, Tunisia; Soltani.abyr@gmail.com (A.S.); joudamediouni1969@gmail.com (J.M.B.J.)

**Keywords:** essential oils, aromatic plants, antifungal, anti-germinative, herbicidal, insecticidal

## Abstract

Owing to their various application fields and biological properties, natural products and essential oils (EO) in particular are nowadays attracting more attention as alternative methods to control plant pathogens and pests, weeds, and for post-harvest applications. Additionally, to overcome EO stability issues and low persistence of effects, EO encapsulation in *β*-cyclodextrin (*β*-CD) could represent a promising avenue. Thus, in this work, the EO distilled from two aromatic plants (*Salvia sclarea* L. and *Coriandrum sativum* L.) have been evaluated in vitro for their antifungal, herbicidal and insecticidal activities, against major plant pathogens and pests of agronomical importance. Both plants were grown on unpolluted and trace-element-polluted soils, so as to investigate the effect of the soil pollution on the EO compositions and biological effects. These EO are rich in oxygenated monoterpenes (clary sage and coriander seeds EO), or aliphatic aldehydes (coriander aerial parts EO), and were unaltered by the soil pollution. The tested EO successfully inhibited the growth of two phytopathogenic fungi, *Zymoseptoria tritici* and *Fusarium* *culmorum*, displaying IC_50_ ranging from 0.46 to 2.08 g L^−1^, while also exerting anti-germinative, herbicidal, repellent and fumigant effects. However, no improvement of the EO biological effects was observed in the presence of *β*-CD, under these in vitro experimental conditions. Among the tested EO, the one from aerial parts of coriander displayed the most significant antifungal and herbicidal effects, while the three of them exerted valuable broad-range insecticidal effects. As a whole, these findings suggest that EO produced on polluted areas can be of great interest to the agricultural area, given their faithful chemical compositions and valuable biological effects.

## 1. Introduction

Historically used in traditional medicine, essential oils (EO) are these days raising great interest, owing to their diverse application fields [1,2,3]. Made of a mixture of volatile compounds, up to 100, and synthesized by all aromatic plant parts as secondary metabolites [4,5], EO were recently outlined for their interest in the preservation of food quality and flavor [6]. They also have received increasing attention as potential alternatives to commercial pesticides in crop protection, given their promising biological properties against plant pathogens, pests and weeds [1,5].

Among fungal phytopathogens, *Fusarium culmorum* and *Zymoseptoria tritici* are of major importance, responsible for *Septoria tritici* blotch and *Fusarium* head blight on cereals, respectively, whose damage on host plant are considerable, and may cause yield losses up to 50% [7,8]. *Fusarium* spp. are also known to produce a wide range of mycotoxins, secondary metabolites that may be highly toxic to human and animal health [8,9]. The control of both *F. culmorum* and *Z. tritici* is mostly achieved through the use of triazole fungicides [8]. However, resistance levels to triazoles have significantly increased since their marketing authorization, and hence compromise their reliability [7,8]. Similarly, insect pests cause significant losses in terms of quantity and quality of the products, in field or during postharvest storage. This is the case for the silverleaf whitefly, *Bemisia tabaci* Genn. (Hemiptera: Aleyrodidae), the lesser grain borer, *Rhyzopertha dominica* F. (Coleoptera: Bostrychidae), or the Mediterranean flour moth, *Ephestia kuehniella* Zeller (Lepidoptera: Pyralidae), who are recognized worldwide as some of the most destructive pests on several economically important crops [10,11,12]. Their control is also mainly achieved through the use of chemical insecticides, displaying resistance phenomena in pest populations [10,12]. Notably, *B. tabaci* has been identified as resistant to a wide number of systemic insecticides, such as organophosphates, synthetic pyrethroids or neonicotinoids [12,13]. Moreover, their use may lead to detrimental effects on beneficial insects, as well [10,14]. From a wider perspective, the excessive and inappropriate use of pesticides is controversial, because of their noxious impact on both environmental and human health [5,15]. As part of an integrated pest management system, the use of natural products, including EO, considered as biocontrol tools, is then greatly encouraged [16,17], especially in the current context, where regulatory restrictions lead to the withdrawal of several commercial products [15,18]. Owing to their relatively low toxicity for humans and animals, EO are moreover registered as “Generally Recognized As Safe (GRAS)” products by the Food and Drug Administration, and regarded as less harmful to both environmental and human health, in comparison with commercial pesticides [19,20]. They also receive increasing public support, considered as eco-friendly products [4,20].

Nevertheless, EO are highly volatile, which could be of great interest for the reduction of the residues, as well as for postharvest applications [4]. Yet, this appears prohibitive for field applications, due to stability issues, and a short persistency of the biological effects over time [5]. To tackle these problems, the use of appropriate EO formulations could offer a promising tool [4,5,21]. In particular, the use of *β*-cyclodextrins (*β*-CD), cyclic oligosaccharides able to encapsulate hydrophobic compounds in aqueous solutions, and cited in the Food and Drug Administration’s list of Inactive Pharmaceutical Ingredients, may be of great interest to avoid degradation, while maintaining the products’ efficiency [19,22].

Within the plant kingdom, Lamiaceae are one of the biggest flowering plant families, comprising a wide number of valuable aromatic species [23,24]. Among them, clary sage (*Salvia sclarea* L.), a biennial aromatic plant species grown all around the world for its high-value EO, has been long known for its use in the perfumery and cosmetic sectors, but also for its applications in medicine [20,25,26]. Coriander (*Coriandrum sativum* L.), another aromatic plant belonging to the Apiaceae family, is an annual herbaceous plant grown all over the world, for the consumption of its green leaves and its seeds, as a spice, or for EO production [27,28]. Both clary sage and coriander EO have drawn attention given their biological effects, in particular antifungal properties against the phytopathogenic fungi *Phoma* spp., *Alternaria alternata* or yeasts, such as *Candida* spp. [26,28,29], and insecticidal effects against the whitefly *Trialeurodes vaporariorum* [26], or several coleoptera species [30,31,32].

In addition to the production of EO, aromatic plants may also appear as valuable choices within the framework of phytomanagement approaches [33,34]. There is in fact an urgent need to address the issue bound to the presence in ecosystems of inorganic pollutants, such as trace elements (TE), whose pollution extent may exceed 5 million sites worldwide [35]. They are posing serious threats to environmental and human health, as they are non-degradable, tend to accumulate in living organisms, and exhibit toxic effects when their concentration exceeds a certain bearable threshold [33,36]. With the emergence of phytotechnologies as rising tools to mitigate TE-polluted spaces, the capacity of some aromatic plants to tolerate elevated concentrations of TE could be particularly valuable [33,34,37]. Their cultivation on marginal lands, unsuitable for food production, tends to minimize the risk of food-chain contamination, while avoiding competition with feeding agriculture [33,34]. Moreover, one of the major drawbacks to phytotechnologies lies in most cases in the lack of economic profitability [38]. In that regard, the use of aromatic plants grown on polluted soils for the production of EO, which are biosourced products bearing a high added-value, and displaying a content free of TE, could help towards the obtention of an economic profit [34,39,40].

Nonetheless, EO composition is strongly influenced by environmental factors, namely geographical location, sunlight, climatic conditions and soil properties, including the presence of pollutants [26,41]. Notably, the presence of TE in soil could result in modified EO yield, and altered composition and quality [42,43,44].

Thus, the aim of this work was first to investigate the potential influence of the soil pollution by TE on the chemical composition of the EO distilled from clary sage inflorescences, and from both aerial parts and seeds of coriander, and then on their biological properties in the presence or in the absence of *β*-CD. The antifungal, anti-germinative and herbicidal potential, as well as the insecticidal activity of the EO, were evaluated against two major phytopathogenic fungi, namely, *F. culmorum* and *Z. tritici*, two plant species commonly used for chemicals’ herbicidal assessments, namely *Lactuca sativa* L. and *Lolium perenne* L., and adults of three insect species, namely, *B. tabaci*, *R. dominica* and *E. kuehniella*, so as to explore their potential use as crop protection products.

## 2. Materials and Methods

### 2.1. Essential Oils

The EO tested in this study were acquired by steam distillation of coriander (*Coriandrum sativum* L.), and clary sage (*Salvia sclarea* L.), two aromatic plant species grown in situ on two experimental sites: a TE-polluted one, displaying elevated amounts of TE (7, 394 and 443 ppm of Cd, Pb, and Zn, respectively), and an unpolluted one. Their full description, as well as plant physiological data, are available in [34,45]. The distillation of the harvested aromatic plant biomass was realized in collaboration with a private EO-distiller. The steam distillation (14 m^3^ distillation unit—saturated water steam, 0.3 bar) was carried out over a three-hour cycle, until no more EO was recovered, under the previously described experimental conditions [20]. Aerial parts of coriander and seeds were harvested at full blossoming, or at seed maturity, respectively, for their distillation. In the same way, clary sage distillation was performed using harvested inflorescences at full blossoming, during its second year of cultivation, when the highest yields are expected [34,46]. EO were stored at 4 °C, in tightly closed brown glass vials, and under modified nitrogen protective atmosphere, until their use.

### 2.2. Determination of the EO Chemical Composition

EO samples were first diluted in ethyl acetate (ratio 1:200 (*v*/*v*)), and then analyzed by electron ionization gas chromatography–mass spectrometry (Shimadzu QP 2010 Ultra), according to the method previously described [20]. Briefly, volatile EO components were separated on a ZB-5MS (Phenomenex—5%-phenyl-arylene/95% dimethylpolysiloxane—10.0 m × 0.10 mm × 0.10 μm) capillary column. The EO solution was then injected in a split mode (0.2 µL; split ratio 1:10). Helium was used as a carrier gas to operate the system, at a constant linear velocity (60 cm s^−1^). The column temperature was held for 2 min at 60 °C, then programmed to linearly increase to 280 °C, at a constant rate of 40 °C min^−1^, and remained at 280 °C for 1 min. 

Mass spectra were recorded within a mass range of 35.0 to 350 (*m*/*z*), at an interface temperature of 280 °C, and an ionisation energy of 70 eV. The EO components were identified by comparison of their retention indices relative to (C8–C30) n-alkanes (Kovats indices), and their obtained mass spectra, with those listed in the NIST (National Institute of Standards and Technology, Gaithersburg, MD, USA), and Wiley 275 computer libraries, as well as those found in the literature [26,27,41,47,48]. Relative percentages of oil constituents were measured from the GC peak areas.

### 2.3. Biological Activities of the EO

#### 2.3.1. Antifungal Activity

##### Phytopathogenic Fungal Strains

Antifungal activities of the different EO were evaluated against two major phytopathogenic fungi—*Fusarium culmorum* and *Zymoseptoria tritici*—by using in vitro assays. *F. culmorum* strain was maintained on PDA (Potato-Dextrose-Agar, Condalab, Spain) medium. The hemibiotrophic fungus *Z. tritici* (strain T02596) was conserved at −80 °C in cryopreservation tubes. Five days prior to the assays, the fungus was cultivated on a PDA medium, in order to produce spores [49].

##### Determination of In Vitro Antifungal Activity against *F. culmorum*

Essential oils’ antifungal activity against *F. culmorum* was evaluated by using an in vitro direct contact assay. It was evaluated according to the method previously described [19,50], with slight modifications. A PDA medium (39 g L^−1^) was first prepared, complemented with 1% (*v*/*v*) DMSO (Thermo Fisher Scientific, Illkirch, France), in which EO were mixed at 50 °C, so as to obtain a final scale of 5 EO concentrations, ranging from 0.005 to 1.0% (*v*/*v*) of EO in the medium. Discs of *F. culmorum* (0.9 cm) were then cut out from the periphery of a 7-day-old fungal colony, and placed at the center of a 9 cm Petri dish containing the PDA medium complemented with EO. The assay was carried out in the absence, and the presence of *β*-CD in the medium, at 10 mM. Mycelium radial growth was measured after a seven-day incubation (20 ± 1 °C). The inhibition rate was calculated following Equation (1):

(1) $$\mathrm{Inhibition}\;\mathrm{rate}\;(\%)=\frac{X_{0}-{\;X}_{i}}{X_{0}}\times 100$$

where X_0_ and X_i_ stand for the average diameter of the fungal colony in control and in treatment, respectively.

Aqueous solutions of DMSO 1% (*v*/*v*) or *β*-CD (10 mM), as well as a marketed fungicide, Aviator XPro (prothioconazole—150 g L^−1^ and bixafen—75 g L^−1^, Stolz, Wailly-Beaucamp, France), were tested as negative and positive controls, respectively. The positive control was evaluated with concentrations ranging from 5 × 10^−5^ to 0.5% (*v*/*v*). Analyses were led in triplicates for each condition.

##### Determination of In Vitro Antifungal Activity against *Z. tritici*

Essential oils’ antifungal activity against *Z. trici* was evaluated by using an in vitro microplate assay, adapted from Fungicide Resistance Committee methods, and similar to the one developed by [4], with a range of eight concentrations (from 0 to 0.8% of EO in the medium) for each and all EO. Briefly, spores of *Z. trici* were collected and placed in a glucose-peptone suspension. The microplates were then inoculated with 60 µL of the calibrated pathogen suspension (2 × 10^5^ spores mL^−1^). The microplates were then incubated under agitation at 110 rpm (20 ± 1 °C), in darkness, with an incubation time of 6 days, determined according to the pathogen’s optimal growth time. The evaluation of its growth was carried out using a spectrometer (620 nm). For each EO concentration, eight replicate wells were used. Additionally, each assay was carried out in triplicate to compare the products. Controls include four non-inoculated wells per EO concentration. Additionally, EO’s effects were assessed in the presence and in the absence of *β*-CD in the culture medium (10 mM). These natural products were compared to a homologated and marketed product: Aviator XPro (prothioconazole—150 g L^−1^ and bixafen—75 g L^−1^), within the same range of concentrations.

##### Determination of In Vitro Antifungal Properties of the EO

The half-maximal inhibitory concentration (IC_50_) of EO (expressed in g L^−1^), required to obtain a fungal pathogens’ growth inhibition of 50%, was calculated for all in vitro assays. A graphical interpolation, complemented with a statistical analysis based on a nonlinear regression, were used to calculate the IC_50_ value [51]. The IC_50_ of each of the tested EO was also classified as either fungicidal or fungistatic, considering its effects. The fungistatic or fungicidal nature of EO was tested by observing growth revival of the inhibited mycelial disc, following its transfer on EO-free PDA medium: no mycelial return to growth defined fungicidal effect, whereas fungistatic effect was characterized by a fungal regrowth capacity on the EO-free medium.

#### 2.3.2. Anti-Germinative and Herbicidal Activities

Inhibitory effects on seedlings’ emergence and growth of the different EO, were assessed against two plant species, *Lolium perenne* L. (monocotyledon) and *Lactuca sativa* L. (dicotyledon), commonly used for chemicals’ herbicidal assessments [52], and listed in the OECD guidelines (2003). An in vitro method was adapted [53,54,55]. EO aqueous solutions (DMSO 1% (*v*/*v*)) were prepared, in the presence or in the absence of *β*-CD (10 mM), and mixed in an agar non-complemented medium (50 °C), then poured into square Petri dishes (120 × 120 mm). EO concentrations used ranged from 5 × 10^−4^ up to 0.5% (*v*/*v*). Seeds were then placed on the solidified agar medium, in sealed Petri dishes, and incubated for 8 days on a day/night cycle, with a 16 h photoperiod (20 ± 1 °C), and an obscurity period of 8 h (16 ± 1 °C). Glyphosate (isopropylamine salt—360 g L^−1^) was used as a positive control within the same concentrations’ range as the tested EO, while aqueous solutions of DMSO 1% (*v*/*v*) or *β*-CD (10 mM) were tested as negative ones.

After the incubation period, germination rates were evaluated by counting germinated seeds, while root elongation was assessed through an imaging software (ImageJ), by measuring root length [55,56]. The analyses were led in triplicates. Graphical interpolation, complemented with a statistical analysis [51], was used to calculate the IC_50_ values, regarding both germination and root elongation parameters.

#### 2.3.3. Insecticidal Activities

##### Insect Individuals

*Bemisia tabaci* adults were collected from a tomato (*Solanum lycopesicum* L.) greenhouse. Adults of *Rhyzoperta dominica* were kept on whole wheat, while *Ephestia kuehniella* adults were reared on wheat flour. Insects were maintained at 25 ± 1 °C and 65 ± 5% relative humidity. Both female and male adult insects were used for bioassays.

##### Repellency Bioassay

Repellency bioassays for the different tested EO were carried out according to the experimental methods previously described [57], at 25 ± 1 °C and 65 ± 5% relative humidity. For that, Whatman filter papers (8 cm diameter) were cut in half. Test solutions were prepared by dissolving 0.4, 1 and 2.5 µL of EO in 1 mL acetone. Each solution was applied to half of the filter paper discs, using a micropipette. The other half of the filter paper was treated with acetone only, as a control. The treated and control half discs were then air-dried under a fan, in order to evaporate the totality of the solvent. Treated and untreated halves were attached to their opposites, using adhesive tape, and placed in Petri dishes. Twenty male and female adult insects were then released at the center of each filter paper disc. Parafilm was used to seal the dishes. Three replicates were used for each concentration, and for each EO. The number of insects in the treated and untreated halves was recorded after 1, 3, 5 and 24 h. Three trials were made for each concentration, and tested by applying the χ^2^ test for homogeneity ratio (1:1). Numbers of *R. dominica*, *E. kuehniela* and *B. tabaci* adults present on both treated and untreated portions of the experimental paper halves were recorded at different times of exposure. Percentage Repellency (PR) was calculated according to the following formula [58]:

(2) Percentage repellency (%)=[Nc−Nt (Nc+Nt)]×100

where Nc and Nt stand for the number of insects on the untreated area and on the treated area, respectively, after various exposure times.

The data were also expressed as RC_50_ values, corresponding to the concentration that repelled 50% of the exposed insects. Three replicates were observed for each EO, at the different exposure times. Replicates were also used for each EO concentration. Comparison was made between the mean number of treated and untreated insects.

##### Fumigation Bioassay

The toxicity of the three EO by fumigation was tested in Plexiglas bottles of 38 mL, in which 10 *R. dominica*, *E. kuehniella* or *B. tabaci* adults were released. Filter paper was cut into 2 cm in diameter pieces, and impregnated with the different EO concentrations 9.09, 22.72 and 56.81 µL L^−1^. Caps were tightly screwed on the vials. Mortality was recorded after 2, 4, 6, 24, 36, 48, 72, 96, 120 and 144 h from the start of exposure. Three replicates were done for each EO, and for each concentration. The control did not show any mortality. Results were expressed as median lethal time (LT_50_), time after which half of a sample population has died, and median lethal concentration (LC_50_), the chemical concentration that results in the death of 50% of a sample population.

### 2.4. Statistical Analyses

Statistical analyses were performed using XLSTAT 2018.1.1 (Adinsoft, Paris, France) software and R 3.6.1 [59]. Before any statistical analysis, Shapiro–Wilk and Bartlett tests were performed to verify normality and homoscedasticity assumptions, respectively. When necessary, non-normal data were “square-root” or “log10” transformed.

Regarding antifungal and herbicidal properties, IC_50_ values resulted from non-linear regression analyses from triplicate assays, and were expressed as mean values and standard deviation (mean ± SD). The comparison of IC_50_ values was carried out using two-way analysis of variance (ANOVA), complemented with a *post-hoc* Tukey-HSD (Honestly Significant Difference) test.

For the insecticidal activity, the statistical analysis was performed using SPSS statistical software, version 20.0. When necessary, data were transformed by common logarithm or exponential, to meet the normality assumptions. All obtained values were the mean of three replications, and were expressed as means ± standard error. For the repellent activity, differences between each EO were tested by one-way ANOVA, followed by Duncan test. From the bioassays data, a Probit analysis was further conducted to estimate RC_50_ on one side, and LC_50_ and LT_50_ values on the other.

## 3. Results

### 3.1. Determination of the EO Chemical Composition

The GC-MS profiles of the three tested EO, from aerial parts or seeds of coriander, and from clary sage inflorescences, are listed in Table 1. In the EO distilled from aerial parts of coriander, 15 compounds were identified, most of which are aliphatic aldehydes, along with some oxygenated monoterpenes. In contrast, coriander seeds and clary sage EO, in which 11 and 22 compounds were identified, respectively, were particularly rich in terpene compounds (Table 1).

The chromatographic profile showed that linalool was the only compound present in the three EO, from different plants, and plant parts. It was particularly abundant in the EO distilled from coriander seeds, with relative proportions ranging between 76.2 and 80.6%, for the EO distilled from the biomass grown on unpolluted and polluted sites, respectively (Table 1). *γ*-terpinene also represented a significant proportion of coriander seeds EO (from 7.8 to 8.7%).

In the EO distilled from coriander aerial parts, the other compounds identified with a proportion higher than 5% were decanal (7.5%) and (Z)-2-decenal (44 to 49.1%), whereas linalyl acetate and germacrene D were the other major compounds in clary sage EO (varying between 52.2 and 62.7%, and between 7.1 and 15.6%, respectively).

It should also be noted that, even though the overall composition was highly similar for a same plant species and part, the balance between several compounds varied slightly, between EO distilled from aromatic plants grown on unpolluted and TE-polluted sites. It is notably the case for linalool, linalyl acetate, and germacrene-D in EO from sage inflorescences, for undecanal, 2-dodecenal, or 2-tridecenal in the EO distilled from aerial parts of coriander, and for *α*-pinene, linalool or *β*-farnesene in the EO from coriander seeds (Table 1).

### 3.2. EO Antifungal Activity

#### 3.2.1. In Vitro Antifungal Activity against *F. culmorum*

**Figure 1:**
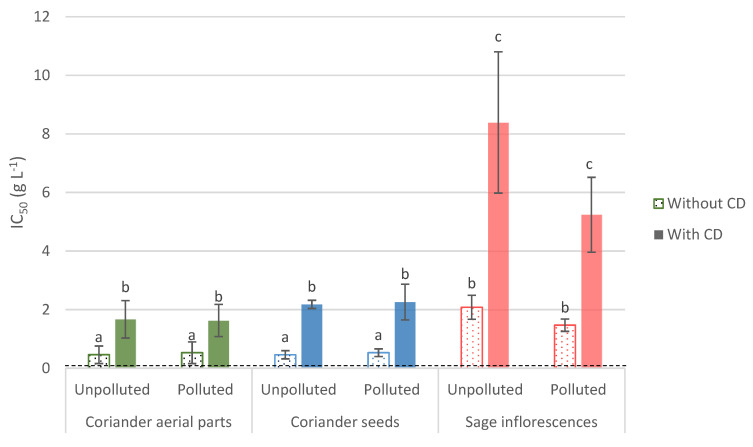
EO’s IC_50_ values (g L^−1^) arising from the antifungal in vitro direct contact bioassay against *F. culmorum*. Values are means ± SD (n = 3). Means followed by the same lowercase letter are not significantly different, by two-way ANOVA comparison (α = 0.05). The positive control (Aviator XPro) value is represented by the black dotted line. All conditions are different from the positive control. IC_50_: half-maximum inhibitory concentration; CD: cyclodextrins.

Our results have shown that the EO from both coriander and sage presented antifungal properties against the phytopathogenic fungus, *F. culmorum*. The results obtained with coriander aerial parts’ EO range of concentrations are provided in Figure S1. It was characterized as fungistatic, since fungal regrowth was observed when the discs containing the fungus were transferred on an EO-free medium. IC_50_ obtained for the direct contact assay ranged from 0.46 to 2.08 g L^−1^, with no significant difference observed between the EO from a same plant, either aerial parts of coriander, seeds of coriander or clary sage, but cultivated under the different experimental conditions (polluted or unpolluted site—Figure 1). Additionally, EO distilled from either aerial parts or seeds of coriander demonstrated a higher efficiency (displaying lower IC_50_) than those from sage, with IC_50_ ranging from 0.46 to 0.53 g L^−1^, and from 1.47 to 2.08 g L^−1^, for coriander and sage, respectively. On another note, no significant improvement was observed in the presence of *β*-CD, displaying either similar or higher IC_50_, in comparison with the *β*-CD-free condition. In comparison with the positive control, all the obtained IC_50_ were significantly higher, up to 104 times (Figure 1).

#### 3.2.2. In Vitro Antifungal Activity against *Z. tritici*

**Figure 2:**
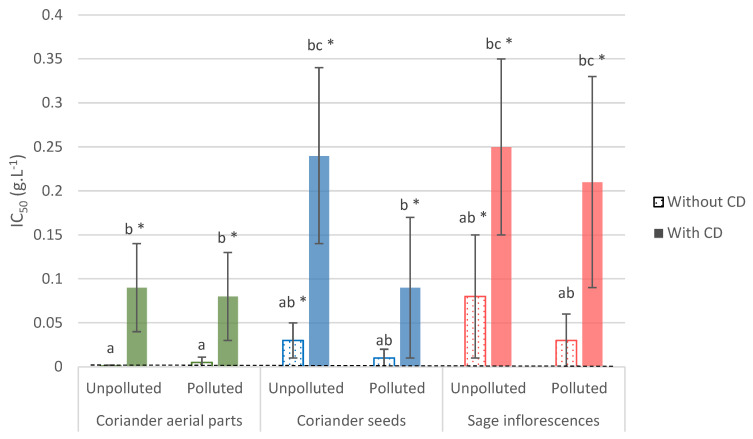
EO’s IC_50_ values (g L^−1^) arising from the antifungal in vitro microplate bioassay against *Z. tritici*. Values are means ± SD (n = 3). Means followed by the same lowercase letter are not significantly different, by two-way ANOVA comparison (α = 0.05). The positive control (Aviator XPro) value is represented by the black dotted line. All conditions different from the positive control are displayed with an asterisk “*”. IC_50_: half-maximum inhibitory concentration; CD: cyclodextrins.

Our results from the microplate assay against *Z. tritici* have shown that the EO from coriander aerial parts or seeds, and sage, presented fungistatic properties against this phytopathogenic fungus. The results obtained for the in vitro microplate bioassay with the three tested EO are provided in Figure S2. IC_50_ obtained for this assay ranged from 0.001 to 0.08 g L^−1^, with no significant difference observed, whatever the EO and the plant part it is distilled from (aerial parts or seeds of coriander, sage inflorescences) and the experimental cultivation conditions (polluted or unpolluted sites—Figure 2). No significant improvement was observed in the presence of *β*-CD, displaying either similar or higher IC_50_ in comparison with the *β*-CD-free condition. In comparison with the positive control, the IC_50_ obtained for both coriander EO (aerials parts or seeds) and sage were similar in our experimental conditions.

### 3.3. EO Anti-Germinative and Herbicidal Activities

#### 3.3.1. Seedlings’ Emergence Inhibition Bioassay

Our results have shown that all tested EO exerted a significant anti-germinative effect on both lettuce and rye-grass. The obtained IC_50_ ranged from 0.05 to 6.22 g L^−1^, and from 0.15 to 9.9 g L^−1^, for EO tested on lettuce, and rye-grass, respectively (Table 2). EO from aerial parts and seeds of coriander have demonstrated a higher efficiency, on both lettuce and rye-grass, than those from sage. Additionally, no difference was found between the EO distilled from a same coriander part, but under the different experimental conditions (polluted or unpolluted soil).

In the presence of *β*-CD, no significant improvement was demonstrated in our experimental conditions, with even significantly higher effects in the absence of *β*-CD for the EO of coriander’s aerial parts, and of sage, on both tested plants (Table 2). Due to the retention of EO by *β*-CD, it sometimes resulted in a very limited efficiency at the tested concentrations. Thus, IC_50_ have not been calculated (NC) for several conditions, as it would have resulted in particularly high and inaccurate values. On another note, in comparison with the positive control, only the EO from aerial parts of coriander are in the same range of efficiency on lettuce, whereas all the tested EO are at least as efficient as the control on rye-grass.

#### 3.3.2. Seedlings’ Growth Inhibition Bioassay

IC_50_ obtained regarding radicle growth inhibition varied from 0.017 to 1.17 g L^−1^, and from 0.050 to 0.66 g L^−1^, for lettuce and rye-grass bioassays, respectively (Table 3). On both lettuce and rye-grass, EO from coriander aerial parts displayed the highest efficiency (Figure S3), in comparison with those from coriander seeds, and from sage. In addition, EO from coriander seeds displayed higher efficiency than those from sage on lettuce. On another note, no difference was visible between the EO distilled from a same plant, but under the different experimental conditions (polluted or unpolluted soil), against both lettuce and rye-grass (Table 3). Additionally, in the presence of *β*-CD, no significant improvement was obtained in our experimental conditions, with even significantly lower effects in the presence of *β*-CD (negative effect), in most cases. In comparison with the positive control, the tested EO displayed IC_50_ more than 100 times higher in the case of both lettuce and rye-grass assays.

### 3.4. EO Insecticidal Activities

**Figure 3:**
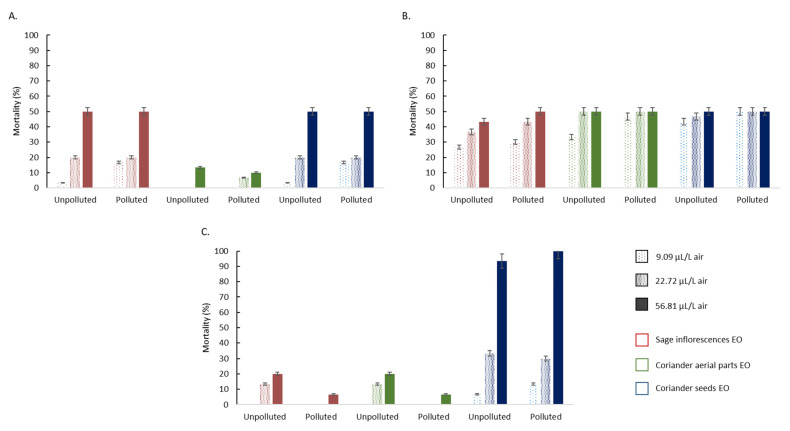
Mortality rates (%) of *E. kuehniella* (**A**), *B. tabaci* (**B**) and *R. dominica* (**C**) adults, exposed during 24 h to three EO (clary sage, coriander aerial parts and coriander seeds) at different concentrations (n = 3).

The mortality rates of *E. kuehniella*, *B. tabaci* and *R. dominica*, exposed for 24 h to different concentrations of clary sage and coriander EO, are presented in Figure 3.

Data related to the effect of the three tested EO against *E. kuehniella* showed that the EO obtained from clary sage and coriander seeds were toxic at the lowest tested concentration (9.09 µL L^−1^ air), displaying mortality percentages ranging from 3.33 to 16.7% (Figure 3A). The mortality percentage significantly increased with higher EO concentrations, to attain 50% at the highest evaluated concentration (56.81 µL L^−1^ air), for both EO. Conversely, the EO from coriander aerial parts did not exert toxic effect at the lowest concentration, while displaying mortality percentages up to 13.3 and 10%, for the unpolluted and polluted conditions, respectively (Figure 3A). According to the mortality percentages, EO toxicity increased in the following order: coriander seeds EO ≤ clary sage EO ≤ coriander aerial parts EO.

These results also demonstrated that the three tested essential oils had toxic effects against *B. tabaci*, even at the lowest tested concentration (9.09 µL L^−1^ air—Figure 3B). The mortality percentages ranged between 26.7 and 50%, at the lowest and highest tested concentrations, respectively. Similar values were obtained between the three tested EO (*p* > 0.05), whatever the condition (polluted or unpolluted site).

Regarding *R. dominica* mortality, the results indicated that no mortality was caused by clary sage, and coriander aerial parts EO, at the lowest concentration (9.09 µL L^−1^ air). Both EO displayed a similar toxicity pattern, with mortality percentages significantly increasing with higher EO concentrations, to attain 20.0 and 6.7% at the highest concentration (56.81 µL L^−1^ air), for the unpolluted and polluted conditions, respectively (Figure 3C). Moreover, lower mortality percentages were obtained with the EO obtained from the plants grown in polluted conditions (F = 72.64; *p* ≤ 0.001). Additionally, in comparison with these 2 EO, significantly higher toxic effects were obtained with the EO from coriander seeds, displaying lethal effects at the lowest tested concentration (ranging from 6.7 to 16.7%), increasing to up to 100% at the highest concentration (56.81 µL L^−1^ air—Figure 3C).

The statistical analysis showed highly significant differences between EO, especially between clary sage or coriander aerial parts EO on one side, and coriander seeds EO on the other, for *R. dominica* (F = 4194.41; *p* ≤ 0.001), and *B. tabaci* (F = 435; *p* ≤ 0.001), or between coriander aerial parts on one side, and clary sage and coriander seeds EO on the other, for *E. kuehniella* (F = 583.65; *p* ≤ 0.001). Moreover, whatever the EO, the concentration had a significant effect on the mortality percentages of the three tested insects, and especially *E. kuehniella* and *R. dominica* (F = 3693.01; *p* ≤ 0.001).

#### 3.4.1. Repellent Activity

The results in terms of EO repellent activity against *E. kuehniella*, *B. tabaci* and *R*. *dominica*, have been evaluated using impregnated filter paper test, and are illustrated in Table 4. Whatever the condition (unpolluted or polluted sites), and the target insect, the three tested EO exhibited an important repellent activity (F = 9.01; *p* ≤ 0.001), ranging from 13.3 to 66.7%, and dependent upon EO, and concentration (*p* < 0.05). In this test, the strongest repellent activity against *E. kuehniella* was caused by the EO distilled from the aerial parts of coriander, at the highest tested concentration (0.1 µL cm^−2^—66.7%), while clary sage EO showed the highest repellent activity against *R. dominica*, up to 63.3% (Table 4). Against *B. tabaci*, the three tested EO revealed similar efficiencies (F = 1.01; *p* = 0.36).

Furthermore, these results showed that the origin of the EO (polluted or unpolluted sites) had no effect on EO repellent potential (F = 1.42; *p* = 0.24), and that the effects of increasing EO concentrations are particularly marked against *E. kuehniella* (F = 65.31; *p* ≤ 0.001) and *B. tabaci* (F = 12.61; *p* ≤ 0.001).

Regarding the median repellent concentrations (RC_50_), the three tested EO showed good repellent activity against the three target insects, displaying RC_50_ values ranging from 2.61 to 3.80 µL cm^−2^, from 2.61 to 3.77 µL cm^−2^, and from 0.07 to 0.16 µL cm^−2^, against *E. kuehniella*, *B. tabaci* and *R. dominica*, respectively (Table S1). It should be noted that *R. dominica* displayed the highest sensitivity to the EO treatments, whatever the EO, and the condition (polluted or unpolluted sites). Furthermore, whatever the insect, the tested EO displayed similar ranges of efficiency, after 24 h of exposure, regardless of the condition (Table S1).

#### 3.4.2. Fumigant Toxicity

The results in terms of fumigant activity were expressed as both median lethal time (LT_50_), and median lethal concentration (LC_50_) values. 

Regarding *E. kuehniella*, the three tested EO exerted a significant activity by fumigation. LC_50_ values ranged between 3.0 and 5.2 µL L^−1^, with mean median lethal times estimated between 87 and 141 h (Table 5). Moreover, no significant difference was observed between LC_50_ values, whatever the EO (*p* > 0.05). However, LC_50_ values were significantly different between the EO distilled from aerial parts of coriander, originating from the unpolluted (3.5 µL L^−1^), and polluted (5.2 µL L^−1^) sites.

Against *B. tabaci*, the data related to LT_50_ values ranged from 25.5 to 37.6 h. All the three tested EO have demonstrated a fumigant lethal potential, at low concentrations ranging from 2.7 to 3.7 µL L^−1^ of EO (Table 6). No significant difference was observed between either LT_50_ or LC_50_ values, whatever the EO, and the plant growing conditions (unpolluted or polluted sites).

Concerning *R. dominica*, LC_50_ values ranged between 2.2 and 4.1 µL L^−1^, while LT_50_ values were measured between 19.8 and 123.1 h (Table 7). No significant difference was observed regarding LC_50_ values, between EO from clary sage inflorescences and aerial parts of coriander (*p* > 0.05), and whatever the plant growing conditions (unpolluted or polluted sites—*p* > 0.05). Nonetheless, the EO distilled from seeds of coriander was more toxic against *R. dominica*, displaying lower LC_50_, ranging between 2.2 and 2.9 µL L^−1^.

As a whole, it should be noted that the three target insects displayed similar ranges of susceptibility to the different EO, tested by fumigation.

## 4. Discussion

Natural compounds from plants, including EO, may be efficient alternatives to the conventional pesticides, especially in integrated approaches. First of all, the possible influence of the soil pollution by TE, on the EO chemical compositions, was assessed.

Then, the potential of EO obtained from clary sage (inflorescences) and coriander (aerial parts and seeds), regarding their antifungal, anti-germinative, herbicidal, and insecticidal activities, was investigated in vitro.

### 4.1. Effect of Soil Pollution on the Chemical Compositions of EO Distilled from Coriander and Clary Sage

In this study, the chemical composition of the EO distilled from coriander aerial parts was characterized by significant proportions of 2-decenal (between 44 and 49%), linalool (up to 35%), decanal, 2-dodecenal, and 2-tridecenal, among 15 different detected aromatic compounds. It is hence mostly composed of aliphatic aldehydes, which is consistent with previous investigations [28,60,61]. Some of these previous reports have shown that the EO chemical composition, and that of coriander EO in particular, was dependent upon the plant part that was used for the distillation [28,60,62]. Thus, it is not surprising that the EO distilled from seeds of coriander displayed a significantly different composition from the one distilled from its aerial parts, and was mostly constituted of monoterpenes, such as α-pinene or *γ*-terpinene, and especially of linalool, up to 81%. These results are consistent with previously published data [60,61]. Clary sage EO mostly consisted of oxygenated monoterpenes, up to 85% of the EO composition, such as linalool, *β*-myrcene, *α*-copaene, or *β*-caryophyllene. Linalyl acetate and linalool, both monoterpenes, are in fact the EO major compounds, as previously reported [20,26,41,48]. Moreover, the rather elevated amount of germacrene-D obtained in our experimental conditions corresponds to a previously described chemotype, rich in that specific compound [26,48,63].

However, even though the EO chemical composition for a same plant and plant part was highly similar between the tested experimental conditions (unpolluted or polluted soils), the relative abundances of several chemical compounds, such as linalool, linalyl acetate, or several aliphatic aldehydes, were found modified. Attention should be drawn to the influence of the environmental parameters on EO composition, such as geographic location, climate, soil conditions, along with cultivation practices [11,48,60]. Notably, the presence of elevated amounts of TE in soil has been shown to result in lower EO yields [43,44,64], or in altered EO chemical compositions, in response to the TE-induced stresses [37,39,65]. It is suspected that in response to TE exposure, inhibition or an activation of several key enzymes—involved in the biosynthesis pathways—could result in a modification of the plant secondary metabolism, and hence lead to either a reduction, or to an increase of specific secondary metabolites, respectively [43,64,65], which could explain the obtained differences. However, the variability among the experimental conditions was rather low, and the quality of the three different EO was faithful according to the chemotypes reported in the literature body [26,28,48,60,61], while the EO yields were in a related publication found unaffected by the soil pollution [34]. Furthermore, as previously highlighted [42,44], the response to TE exposure seems to vary greatly among aromatic plant species. In that regard, the chemical composition from clary sage inflorescence seemed to be less affected than the EO from coriander by the environmental conditions, and in particular by the presence of TE in soil. Aromatic plants from the genus *Salvia* (Lamiaceae) in particular, were in fact described as being able to tolerate elevated TE amounts, and to consistently grow in such conditions, displaying unaffected EO compositions [40,42], corroborating the obtained results. Finally, from a wider perspective, the variability recorded in terms of EO composition could be attributed not only to the presence of TE in soil, but also to the geographical location, and to the soil conditions [48,60]. In previously published data [34,45], it was indeed highlighted that the soil physico-chemical parameters were slightly different between the two experimental sites, which could explain that the two aromatic plants grown in situ displayed slightly different EO chemical compositions, even though the plant maturity stages at harvest were identical, and that TE in soil did not hinder plant growth [34].

### 4.2. EO Biological Activities towards a Potential Application in Crop Protection

EO from both coriander and clary sage were previously investigated for their antifungal activity against a wide spectrum of fungal pathogens, but reports targeting plant pathogens in particular are scarce. Until now, positive results have been reported regarding the antifungal effects of EO from coriander seeds, and from Lamiaceae species, on the development of *Fusarium* spp. or various other fungal phytopathogens [4,60]. As highlighted in the majority of the previous studies, EO biological effects are often dependent upon the EO concentration [4]. In the same way, our data regarding antifungal activity, against both *F. culmorum* and *Z. tritici*, have shown that all the EO that were evaluated in this study inhibited fungal growth. Notably, the observed antifungal effect was defined as fungistatic rather than fungicidal, depicted by the revival of hyphae and mycelial growth, after transfer on a medium exempted from EO. This feature could be valuable in preventive applications as a means to control pre- and postharvest fungal diseases. The efficiency of the EO increased in the following order: clary sage EO < coriander seeds EO ≤ coriander aerial parts EO. Although the concentrations of the EO were up to 1000 times higher than those of the chemical marketed fungicides, which is commonly observed [66], the tested EO still displayed consistent antifungal activity. Furthermore, EO are known to exert lower harmful effects on non-target organisms, and on environment and human health [4,11]. Notably, they are known to possess a low persistence in soils, owing to their volatility [4,67,68,69], while the occurrence of resistance phenomena bound to the use of EO has not been reported so far. This feature could be bound to their action as multisite chemicals [4]. It should also be noted that, in the case of *Z. tritici*, all the tested EO were in the same range of efficiency as the positive control consisting in a marketed fungicidal product. This feature could be particularly interesting. Indeed, by displaying a substantial biocidal activity, combined with a limited toxicity towards non-target organisms, and a high volatility hence limiting environmental risks, the tested EO appear as promising candidates, when compared to conventional pesticides or even other biocontrol products.

In addition to antifungal properties, the tested EO revealed a significant anti-germinative effect, and herbicidal activity, on both lettuce and rye-grass. Our results depicted a promising activity of the EO, especially the one distilled from aerial parts of coriander, which displayed lower IC_50_ values than those from coriander seeds and clary sage, whatever the bioassay, and the target plant. In comparison with glyphosate, which is a systemic herbicide, and was evaluated as a positive control in this work, the tested EO displayed a consistent herbicidal activity. In previous studies, reported glyphosate IC_50_ varied from 15.3 mg L^−1^ [70] to 23 and 46.2 mg L^−1^ [71] regarding the inhibition of ryegrass growth, while the results reported on lettuce ranged from 8.9 mg L^−1^ [72] to 20 mg L^−1^ [73], which are comparable to those obtained in this study. In addition, the EO from aerial parts of coriander, reported as the most efficient in terms of in vitro herbicidal activity, exerted effects similar to those of glyphosate, and even higher on rye-grass. Since glyphosate is not homologated as an anti-germinative product, the use of EO to fulfil this purpose could be promising.

### 4.3. EO Potential Applications as Post-Harvest Pests Control Products—Insectidical Properties

Secondary metabolites from plants are also recognized to play a role in plant–insect interactions, and as such have been widely investigated for their insecticidal properties [26,74]. Their quick degradation could also favor their use as fumigants [11,75].

In this study, the potent repellent and fumigant activities were examined against the adults of *E. kuehniella*, *B. tabaci* and *R. dominica*.

In response to EO exposure, *E. kuehniella* and *B. tabaci* displayed mortality percentages up to 50%, whatever the EO, while coriander seeds EO displayed a mortality rate up to 100% against *R. dominica*. Moreover, whatever the EO concentration, the three tested EO displayed a similar range of efficiency against *B. tabaci*—the EO, and in particular those from coriander, resulted in a substantial insect mortality, even at low concentrations. Regarding repellence, the three tested EO displayed similar efficiencies against *B. tabaci*, while the EO from clary sage and from the aerial parts of coriander displayed the highest repellence percentages against *R. dominica* and *E. kuehniella*, respectively. Moreover, the obtained RC_50_ values ranged between 2.61 and 3.80 µL cm^−2^, and between 2.61 and 3.77 µL cm^−2^, against *E. kuehniella*, and *B. tabaci*, respectively, while those obtained against R. *dominica* were significantly lower, varying from 0.07 to 0.16 µL cm^−2^. Finally, in the current fumigant bioassays, the three tested EO showed similar ranges of toxicity against the three target insects, ranging from 2.2 to 5.2 µL L^−1^.

The insecticidal activity of several plant extracts and EO has previously been reported in several studies [26,66,67,74,75]. Little work has however been done using coriander or clary sage EO against the insects that are targeted in the present study. 

Against *B. tabaci*, diverse EO were previously evaluated, such as those from *Citrus aurantium peels*, *Citrus sinensis*, *Allium sativum*, *Agastache rugosa*, *Illicium verum*, *Chenopodium ambrosioides*, *Schizonepeta tenuifolia*, *Curcuma aeruginosa*, *Syzygium aromaticum* or *Valeriana officinalis* [12,76,77]. Among all the tested EO, the strongest fumigant activities were obtained with the EO from *A. sativum*, *C. aurantium* and *A. rugosa*, with respective LC_50_ values of 0.11 µg L^−1^, 3.97 and 5.8 µL L^−1^ and 7.08 µg L^−1^ [12,76,77]. In contrast, some EO did not result in any fumigant toxicity at the tested concentrations, such as those from *S. tenuifolia*, *C. aeruginosa* or *V. officinalis* [76]. Whatever the bioassay, the three EO tested in the current study displayed a fumigant activity, and similar efficiencies against *B. tabaci*. Moreover, in comparison with the body of literature, the obtained LC_50_ values would put them among the most efficient EO reported so far against *B. tabaci*. 

Concerning *E. kuehniella*, EO from *Ocimum basilicum*, *Mentha pulegium* or *Ruta graveolens* previously displayed LC_50_ values ranging from 0.3 to 1.02 µL L^−1^ [11], while the one from *Pistacia lentiscus* was about 40.2 µL L^−1^ [11,78]. Essential oils from *Eucalyptus astringens*, *Eucalyptus leucoxylon*, *Eucalyptus lehmannii*, *Eucalyptus rudis*, *Eucalyptus camaldulensis*, and *Laurus nobilis* were also effective against *E. kuehniella*, since the related LC_50_ values ranged between 20.5 and 33.8 µL L^−1^ [11,79,80]. Thus, the results obtained during the present investigation suggest good potential for the three tested EO to be used as both fumigant and repellent products.

Coriander seeds EO, as well as its isolated major compounds, were previously evaluated against *R. dominica* [81]. A high mortality rate after 24 h of exposure (up to 100%) was observed, using a dose of 1 µL/15 mL of EO, which corroborates the high mortality percentages obtained with the EO distilled from seeds of coriander in the current experimental conditions. The EO from seeds of coriander has also been evaluated against several stored products pests, such as *Tribolium castaneum*, *Lasioderma serricorne* and *Sitophilus oryzae* [82], while the one distilled from coriander aerial parts was investigated for its effects against *T. castaneum* [83]. High inhibition of *T. castaneum* early development stages was observed [83], along with a significant fumigant toxicity reflected in LC_50_ values of 276.3, 5.3, and 145.5 µL L^−1^ of air, against *T. castaneum*, *L. serricorne* and *S. oryzae*, respectively [82]. The LC_50_ values acquired for the EO of clary sage, and coriander seeds and aerial parts, are this way within the same range of efficiency, and among the most efficient ones. 

Against *R. dominica*, formulated aqueous extracts of clary sage were also previously reported for their toxic effects, with mortality rates above 95%, at the highest tested concentration [84]. These mortality rates, higher than those obtained in the present study, highlight the importance of an adequate EO formulation, so as to improve the EO biological effects as well as their persistence in time, often brought forward as limited [84,85,86].

### 4.4. Essential Oil Encapsulation in β-CD

To address this issue, EO encapsulation in cyclic oligosaccharides, such as *β*-CD, could help preventing EO oxidation, thermal degradation and quick evaporation, and allow a controlled-release of the EO and of their major compounds [87,88,89,90]. Interestingly, the EO studied in this work were previously demonstrated to be efficiently complexed with *β*-CD, since retention percentages ranged from 63 to 80% [20]. These are within the same range as those commonly described for some other EO [20,86]. It hence suggests that CD can efficiently retain EO and further reduce their volatility. However, in our experimental conditions, EO complexation with *β*-CD did not result in a significant improvement of the investigated biological properties. In some specific cases, notably in the antifungal and herbicidal assays, it even resulted in a lower efficiency of the EO (negative effect), owing to their complexation with *β*-CD and consequently their reduced volatility and availability. From an agricultural perspective, encapsulation could nonetheless significantly raise the persistence of the EO’s effects, given their efficient retention by *β*-CD, allowing a controlled release [89,91]. It could particularly be valuable towards a lengthening of the fungistatic effects in time, which could then be of great interest to legitimize their use as natural alternatives.

Overall, our results suggest that the presence of TE in soil did not alter the EO biological effects, whatever the assessed property. Whether they were evaluated for applications in crop protection or as post-harvest treatments, EO originating from the biomass cultivated on the polluted site mostly displayed similar efficacies as the one distilled from the unpolluted one.

### 4.5. Insights on the Relationships between EO Composition and Their Biological Effects

Mono- and sesquiterpenoids are commonly described as responsible for the EO biological activities, whether they are antimicrobial, herbicidal or insecticidal [12,28,81,92,93]. As such, linalool, camphor and geranyl acetate were highlighted as the active compounds of the EO distilled from seeds of coriander, in terms of fumigant toxicity against *R. dominica* [82]. Similarly, clary sage EO insecticidal activity could be bound to its high amount of linalyl acetate in particular, since the exclusion of that compound from the EO mixture resulted in a substantial decrease in terms of repellence (halved) against a mite species, *Tetranychus urticae* [31]. From a wider perspective, linalool which is present in all the three tested EO, but in different proportions, is often highlighted as one of the main factors responsible for the EO bioactivity [81,94]. However, the variation observed between the different tested EO, whatever the biological property, cannot be explained by the action of their major components only.

In fact, it has been repeatedly emphasized that EO’s biological effects were rather the result of a synergism between their compounds, since the evaluation of the latter isolated or of the mixture purified from one of its compounds, resulted in lower activities [12,28,95,96,97]. Since EO could act as multisite chemicals, lowering the risk of resistance phenomena [4], a deeper knowledge of their action mechanisms, and of some of their compounds, alone or in combination, would be of great interest. Even though the biological properties of a wide number of EO against various pathogenic microorganisms and pests have been covered, the investigation of the action mechanisms remains indeed limited. Several main features have nonetheless been highlighted regarding antifungal activity, such as the inhibition of the fungal cell wall formation, the disruption of the cell membrane (through the inhibition of the ergosterol synthesis), the inhibition of the mitochondrial electron transport, the inhibition of the cellular division, the interference with RNA, DNA synthesis and/or protein synthesis, and the inhibition of efflux pumps [5,98,99]. In that regard, coriander EO was demonstrated as efficient against *Candida albicans*, by increasing membrane permeability through a binding interaction with a membrane ergosterol [28,100]. Insecticidal activity of the EO, which has also been thoroughly investigated, points towards a site of action in the insect nervous system [66]. Plants’ EO, and especially terpenoids compounds in it, seem to exhibit their toxicity through an interaction with different putative receptors, namely acetylcholinesterase, nicotinic acetylcholine receptor, octopamine receptor, or gamma-aminobutyric acid receptor ion channel [66,101]. They could moreover target multiple sites simultaneously [66], and act as insect repellents [81]. Regarding EO phytotoxic effects, resulting in visible symptoms, they can notably be the result of mitosis inhibition, a decrease of cellular respiration, ion leakage, membrane depolarization, decrease of the chlorophyll content, oxidative damages or removal of the cuticular waxy layer [5,102,103,104]. In the case of cinnamon and Java citronella EO, or of their main compounds, which could act as efficient herbicides, it was, for instance, demonstrated that the plant plasma membrane could be one of the EO’s cellular targets, owing to the amphiphilic nature of several compounds [102]. The authors concluded that the mentioned EO or compounds were susceptible to affect lipid organization and/or domain formation, especially in the case of monoterpenes, while phenylpropanoids are likely to interact with membrane receptors [102]. However, no comprehensive study has so far been carried out on the detailed herbicidal mechanisms [102], which could be a valuable addition to the field.

## 5. Conclusions

The growing number of studies related to EO biological effects tends to demonstrate their suitability for the development of natural products-based biopesticides [4,17], provided that EO stability issues are solved. Our results demonstrate that growing aromatic plant on TE-polluted surfaces—and distilling EO from the grown biomass—could be a relevant tool to engage the reclaiming of these marginal lands. 

As a whole, the obtained results indicate that the three evaluated EO, from coriander (aerial parts and seeds) and clary sage (inflorescences), displayed faithful chemical compositions, despite the soil pollution by TE. They also were able to inhibit the growth of two major fungal phytopathogens, while also exerting anti-germinative and herbicidal effects, against both mono- and dicotyledon species. Notably, the EO distilled from aerial parts of coriander possessed a higher efficiency, whatever the tested biological activity. Interestingly, significant repellent and fumigant activities were also demonstrated against three major post-harvest pests, whatever the EO. As a result, these EO could be promising candidates for the development of new biopesticides. Nonetheless, if such in vitro assays may indicate the EO’s potential towards applications in crop protection or as post-harvest decay control products, these effects need to be confirmed by further *in planta* or in vivo assays, so as to legitimate their use. Moreover, even though the encapsulation of the tested EO in *β*-CD did not result in any improvement of the biological properties, further assessments should be conducted to confirm the efficiency of the controlled release of EO in glasshouse or field conditions.

Furthermore, these EO could be tested in combination with conventional marketed products, as well as with other EO or biocontrol products, so as to reduce the amounts used, or investigate potential synergistic effects.

## Figures and Tables

**Table 1:** Chemical composition of the EO from aerial parts or seeds of coriander, and from sage inflorescences, grown on unpolluted or TE-polluted sites. Data are relative percentages of EO compounds, expressed as means ± SD (n = 3). For a same plant part, means followed by an asterisk “*” are significantly different, between polluted and unpolluted conditions, by one-way ANOVA test (α = 0.05).

Experimental Retention Indexes	EO Compounds	Aerial Parts of Coriander		Seeds of Coriander		Sage Inflorescences	
		Unpolluted	Polluted	Unpolluted	Polluted	Unpolluted	Polluted
908	*α*-pinene	1.2 ± 0.2	1.2 ± 0.2	4.8 ± 0.5 *	3.1 ± 0.3	-	-
944	Camphene	-	-	0.5 ± 0.1	0.4 ± 0.1	-	-
991	*β*-myrcene	-	-	-	-	1.7 ± 0.3	1.1 ± 0.2
1005	4-carene	-	-	0.1 ± 0.2	0.3 ± 0.5	0.1 ± 0.1	0.1 ± 0.1
1027	Limonene	-	-	1.6 ± 0.1	1.6 ± 0.2	0.3 ± 0.5	0.2 ± 0.2
1034	p-cymene	0.3 ± 0	0.5 ± 0.5	1.6 ± 0	1.3 ± 0.1	-	-
1040	*β*-phellandrene	-	-	-	-	0.1 ± 0.1	0.1 ± 0
1049	Ocimene	-	-	-	-	0.6 ± 0.1	0.6 ± 0.2
1065	*γ*-terpinene	1.7 ± 0.2	1.7 ± 0.2	8.7 ± 0.2	7.8 ± 1	-	-
1100	Linalool	26.8 ± 4.3	34.5 ± 4.1	76.2 ± 1	80.6 ± 2.3 *	10.3 ± 0.2	15.4 ± 1 *
1133	Camphor	1.2 ± 0.2	1.5 ± 0.2	3.6 ± 0.1	3.7 ± 0.1	-	-
1193	*α*-terpineol	-	-	-	-	1.7 ± 0.3	2 ± 0.3
1205	Decanal	7.5 ± 0.8	7.5 ± 0.5	-	-	-	-
1250	Linalyl acetate	-	-	-	-	52.2 ± 1.4	62.7 ± 0.2 *
1274	(Z)-2-decenal	49.1 ± 2	44 ± 5.3	-	-	-	-
1308	Undecanal	-	1.8 ± 1.5 *	-	-	-	-
1371	2-undecenal	1.4 ± 0.3	0.8 ± 0.7	-	-	-	-
1375	*α*-copaene	-	-	-	-	3.9 ± 0.5 *	2 ± 0.1
1383	Geranyl acetate (cis)	-	-	-	-	1 ± 0.2	0.9 ± 0.2
1386	Geranyl acetate (trans)	-	-	2.1 ± 0.8	1.3 ± 0.9	2.2 ± 0.2	2.2 ± 0.6
1389	*β*-cubebene	-	-	-	-	0.1 ± 0.1	0.1 ± 0
1414	*β*-caryophyllene	-	-	-	-	3.4 ± 0.4	2.4 ± 0.3
1420	Dodecanal	0.6 ± 0.1	0.8 ± 0	-	-	-	-
1427	*β*-copaene	-	-	-	-	1.2 ± 0.2	0.6 ± 0.1
1447	*β*-farnesene	1.4 ± 0.1 *	0.2 ± 0.2	0.3 ± 0.5 *	-	0.03 ± 0.1	0.03 ± 0.1
1467	2-dodecenal	5 ± 0.7 *	3.5 ± 0.4	-	-	-	-
1479	Germacrene D	-	-	-	-	15.6 ± 1.3 *	7.1 ± 0.3
1484	*α*-Humulene	-	-	-	-	0.4 ± 0	0.2 ± 0.1
1515	Tridecanal	0.3 ± 0.5 *	-	-	-	-	-
1523	*β*-cadinene	-	-	-	-	1.3 ± 0.2	0.5 ± 0.1
1551	Germacrene B	-	-	-	-	1.8 ± 0.2	0.9 ± 0
1570	2-tridecenal	3.7 ± 0.2 *	2.3 ± 0.2	-	-	-	-
1580	Caryophyllene oxide	-	-	0.1 ± 0.1 *	-	0.3 ± 0.1	0.2 ± 0
1900	Sclareol oxide	-	-	-	-	0.5 ± 0.1	0.4 ± 0.1
2220	Sclareol	-	-	-	-	0.3 ± 0	0.3 ± 0.1

“-”: undetected compound.

**Table 2:** EO’s IC_50_ (g L^−1^) resulting from the seedlings’ emergence inhibition bioassay against lettuce and rye-grass. Values are means ± SD (n = 3).

Bioassay		Aerial Parts of Coriander		Seeds of Coriander		Sage Inflorescences		Positive Control
		Without *β*-CD	With *β*-CD	Without *β*-CD	With *β*-CD	Without *β*-CD	With *β*-CD	
**Lettuce**	Unpolluted	0.05 ± 0.01 a	603 ± 138 b	0.56 ± 0.12 c	1.87 ± 0.17 d	6.22 ± 2.72 e	NC	0.014 ± 0.006 a
	Polluted	0.08 ± 0.01 a	604 ± 107 b	0.73 ± 0.24 c	1.86 ± 0.02 d	4.21 ± 1.08 e	NC	
**Rye-grass**	Unpolluted	0.15 ± 0.03 a’	500 ± 317 b’	0.60 ± 0.06 c’	1.73 ± 0.26 d’	2.6 ± 0.39 d’	3093 ± 975 f’	36.5 ± 15.1 e’
	Polluted	0.16 ± 0.05 a’	782 ± 679 b’	0.74 ± 0.13 c’	1.80 ± 0.26 d’	9.9 ± 8.3 e’	1273 ± 555 f’	

IC_50_: inhibitory concentration; NC: not calculable; Positive control: glyphosate. The different letters are the result from a two-way ANOVA comparison (α = 0.05), between results obtained in free *β*-CD condition, and in the presence of *β*-CD. Means followed by the same letter—without and with apostrophe for lettuce and rye-grass assays, respectively—do not significantly differ.

**Table 3:** EO’s IC_50_ (g L^−1^) resulting from the growth inhibition bioassay against lettuce and rye-grass. Values are means ± SD (n = 3).

Bioassay		Aerial Parts of Coriander		Seeds of Coriander		Sage Inflorescences		Positive Control
		Without *β*-CD	With *β*-CD	Without *β*-CD	With *β*-CD	Without *β*-CD	With *β*-CD	
**Lettuce**	Unpolluted	0.017 ± 0.001 a	0.31 ± 0.07 b	0.28 ± 0.05 b	2.09 ± 0.14 c	1.16 ± 0.45 c	3.96 ± 2.89 c	0.0001 ± 0.0001 d
	Polluted	0.028 ± 0.010 a	0.19 ± 0.08 b	0.29 ± 0.07 b	1.90 ± 0.36 c	1.17 ± 0.56 c	2.49 ± 0.50 c	
**Rye-grass**	Unpolluted	0.050 ± 0.014 a’	0.84 ± 0.40 b’	0.25 ± 0.07 c’	1.93 ± 0.33 d’	0.66 ± 0.14 bc’	3.09 ± 2.2 d’	0.0016 ± 0.0006 e’
	Polluted	0.053 ± 0.010 a’	0.94 ± 0.80 b’	0.25 ± 0.04 c’	1.41 ± 0.24 d’	0.50 ± 0.04 bc’	2.52 ± 1.52 d’	

IC_50_: inhibitory concentration; NC: not calculable; Positive control: glyphosate. The different letters are the result from a two-way ANOVA comparison (α = 0.05), between results obtained in free *β*-CD condition, and in the presence of *β*-CD. Means followed by the same letter—without and with apostrophe for lettuce and rye-grass assays, respectively—do not significantly differ.

**Table 4:** Percentage repellency of the three different EO (sage and aerial parts and seeds of coriander), from the two experimental plots—after 24 h of exposure—against *E. kuehniella*, *B. tabaci* and *R. dominica* adults. Values are means ± SE (n = 3).

Insect Species		Aerial Parts of Coriander		Seeds of Coriander		Sage Inflorescences	
		Unpolluted	Polluted	Unpolluted	Polluted	Unpolluted	Polluted
***E. kuehniella***	0.016 µL cm^−2^	13.3 ± 0.6	13.3 ± 1.1	10.0 ± 1.3	12.5 ± 0.9	6.7 ± 1.1	10.0 ± 0.6
	0.04 µL cm^−2^	26.7 ± 5.8	36.7 ± 3.1	28.9 ± 10.2	18.9 ± 1.7	26.7 ± 2.0	33.4 ± 2.8
	0.1 µL cm^−2^	66.7 ± 7.9	46.7 ± 11.5	50.0 ± 9.6	42.2 ± 11.8	43.4 ± 1.8	46.7 ± 0.3
***B. tabaci***	0.016 µL cm^−2^	13.3 ± 1.6	13.3 ± 0.7	6.7 ± 2.1	13.3 ± 0.5	6.8 ± 0.5	6.8 ± 1.7
	0.04 µL cm^−2^	20.0 ± 4.6	26.7 ± 3.1	20.0 ± 0	26.7 ± 5.7	26.7 ± 0.8	26.7 ± 2.1
	0.1 µL cm^−2^	40.0 ± 6.7	53.3 ± 5.8	40.0 ± 5.0	40.0 ± 5.7	40.0 ± 2.5	53.3 ± 3.1
***R. dominica***	0.016 µL cm^−2^	30.0 ± 1.7	16.7 ± 1.3	16.7 ± 3.4	13.3 ± 4.9	16.7 ± 1.7	16.7 ± 4.0
	0.04 µL cm^−2^	23.3 ± 4.8	23.3 ± 4.9	23.3 ± 0.7	30.0 ± 4.1	16.7 ± 2.9	53.3 ± 3.3
	0.1 µL cm^−2^	33.4 ± 3.3	33.3 ± 4.7	36.7 ± 2.2	43.3 ± 2.3	63.3 ± 2.6	56.7 ± 0.9

**Table 5:** LT_50_ (h) and LC_50_ (µL L^−1^) values resulting from the fumigation bioassay against *E. kuehniella* adults, for the three different tested EO, originating from the two experimental plots (n = 3).

Distilled Plant Part		Concentration (µL L^−1^)	LT_50_ (h)	LC_50_	χ^2^	Slope ± SE	*p*
**Sage inflorescences**	Unpolluted	9.09	101.6	3.0	0.24	0.9 ± 0.1	0.02
		22.72	99.8				
		56.81	121.0				
	Polluted	9.09	123.5	3.2	4.40	0.5 ± 0.1	0.04
		22.72	97.4				
		56.81	41.2				
**Aerial parts of coriander**	Unpolluted	9.09	121.8	3.5	0.51	2.2 ± 1.4	0.01
		22.72	113.3				
		56.81	187.7				
	Polluted	9.09	136.9	5.2	3.41	0.5 ± 0.03	0.01
		22.72	136.2				
		56.81	131.3				
**Seeds of coriander**	Unpolluted	9.09	135.5	3.5	11.80	0.7 ± 0.1	0.01
		22.72	103.2				
		56.81	102.4				
	Polluted	9.09	104.2	3.8	1.49	0.5 ± 0.02	0.01
		22.72	126.8				
		56.81	102.8				

LT_50_: median lethal time; LC_50_: median lethal concentration; SE: standard error.

**Table 6:** LT_50_ (h) and LC_50_ (µL L^−1^) values resulting from the fumigation bioassay against *B. tabaci* adults, for the three different tested EO, originating from the two experimental plots (n = 3).

Distilled Plant Part		Concentration (µL L^−1^)	LT_50_ (h)	LC_50_	χ^2^	Slope ± SE	*p*
**Sage inflorescences**	Unpolluted	9.09	37.6	3.7	0.16	0.1 ± 0.1	0.01
		22.72	33.9				
		56.81	35.4				
	Polluted	9.09	36.2	2.9	0.30	0.3 ± 0.1	0.01
		22.72	29.4				
		56.81	32.8				
**Aerial parts of coriander**	Unpolluted	9.09	36.2	2.7	2.03	0.2 ± 0.1	0.01
		22.72	27.1				
		56.81	25.5				
	Polluted	9.09	31.6	2.7	0.06	0.04 ± 0.01	0.04
		22.72	28.4				
		56.81	26.5				
**Seeds of coriander**	Unpolluted	9.09	36.0	3.6	0.01	0.3 ± 0.1	0.05
		22.72	27.0				
		56.81	25.5				
	Polluted	9.09	29.4	2.7	0.11	0.1 ± 0.01	0.05
		22.72	25.5				
		56.81	25.5				

LT_50_: median lethal time; LC_50_: median lethal concentration; SE: standard error.

**Table 7:** LT_50_ (h) and LC_50_ (µL L^−1^) values resulting from the fumigation bioassay against *R. dominica* adults, for the three different tested EO, originating from the two experimental plots (n = 3).

Distilled Plant Part		Concentration (µL L^−1^)	LT_50_ (h)	LC_50_	χ^2^	Slope ± SE	*p*
**Sage inflorescences**	Unpolluted	9.09	84.8	4.1	5.73	0.7 ± 0.2	0.01
		22.72	60.4				
		56.81	123.1				
	Polluted	9.09	108.5	3.8	0.05	2.0 ± 1.6	0.04
		22.72	68.6				
		56.81	11.2				
**Aerial parts of coriander**	Unpolluted	9.09	84.8	4.1	6.04	0.7 ± 0.2	0.02
		22.72	60.4				
		56.81	123.1				
	Polluted	9.09	108.5	3.7	0.05	2.0 ± 1.7	0.03
		22.72	68.6				
		56.81	118.7				
**Seeds of coriander**	Unpolluted	9.09	19.8	2.2	5.69	1.5 ± 0.1	0.01
		22.72	100.0				
		56.81	97.5				
	Polluted	9.09	103.1	2.9	6.87	0.2 ± 0.1	0.01
		22.72	84.2				
		56.81	62.9				

LT_50_: median lethal time; LC_50_: median lethal concentration; SE: standard error.

## Data Availability

The original contributions presented in the study are publicly available.

## Supplementary Materials

The following supporting information can be downloaded at: https://www.mdpi.com/article/10.3390/foods11030312/s1, Figure S1: Inhibitory effect of increasing EO concentrations from aerial parts of coriander, on the mycelial growth of *F. culmorum*, incubated for seven days, Figure S2: Antifungal activity of the three tested EO (aerial parts of coriander, a; seeds of coriander, b; and clary sage, c) against *Z. tritici*. Results for the in vitro microplate assay are displayed as optical densities—means from 4 values per well, Figure S3: Herbicidal activity of increasing EO concentrations from aerial parts of coriander, against *L. perenne* (a) and *L. sativa* (b), Table S1: RC_50_ values (µL cm^−2^) for the three different tested EO, from the two experimental plots—after 24 h of exposure—against *E. kuehniella*, *B. tabaci* and *R. dominica* adults (n = 3).
