# Supplementary material for: In Vitro Potential of Clary Sage and Coriander Essential Oils as Crop Protection and Post-Harvest Decay Control Products
Source: Foods. 2022 Jan 24;11(3):312. doi: 10.3390/foods11030312 (PMC8834200; doi:10.3390/foods11030312)
Supplement: Supplementary file 1 [file foods-11-00312-s001.zip › Supplementary Table S1.pdf]

Table S1. RC_{50} values (µl.cm^{-2}) for the three different tested EO, from the two experimental plots – after 24 h of exposure – against *E. kuehniella*, *B. tabaci*, and *R. dominica* adults (n = 3).

Insect species		Aerial parts of coriander		Seeds of coriander		Sage inflorescences	
		Unpolluted	Polluted	Unpolluted	Polluted	Unpolluted	Polluted
E. kuehniella	RC_{50}	3.80	3.70	2.61	3.31	3.01	3.80
	Slope ± SE	0.51 ± 0.11	0.51 ± 0.10	0.55 ± 0.01	0.71 ± 0.11	0.66 ± 0.11	0.52 ± 0.11
	χ^2	0.45	0.50	0.71	0.08	4.40	0.45
B. tabaci	RC_{50}	3.77	2.63	2.61	2.61	3.14	3.19
	Slope ± SE	0.46 ± 0.1	0.84 ± 0.1	0.55 ± 0.09	0.55 ± 0.09	0.7 ± 0.09	0.38 ± 0.08
	χ^2	0.52	1.24	0.7	0.72	0.08	0.38
R. dominica	RC_{50}	0.08	0.07	0.16	0.11	0.10	0.13
	Slope ± SE	31.6 ± 2.78	-4.8 ± 2.06	9.73 ± 2.5	12.19 ± 2.3	15.95 ± 2.37	10.9 ± 2.39
	χ^2	8.46	5.28	0.23	40.37	2.12	3.77

RC_{50}: median repellent concentration; SE: Standard error. Data have been tested by χ2-test for homogeneity of 1:1 ratio.
