# Supplementary figures and images for: In Vitro Potential of Clary Sage and Coriander Essential Oils as Crop Protection and Post-Harvest Decay Control Products
Source: Foods. 2022 Jan 24;11(3):312. doi: 10.3390/foods11030312 (PMC8834200; doi:10.3390/foods11030312)

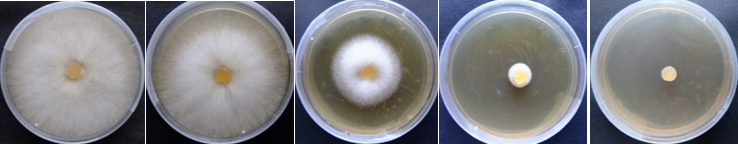

Supplement: Supplementary file 1 [file foods-11-00312-s001.zip › Figure S1.png]

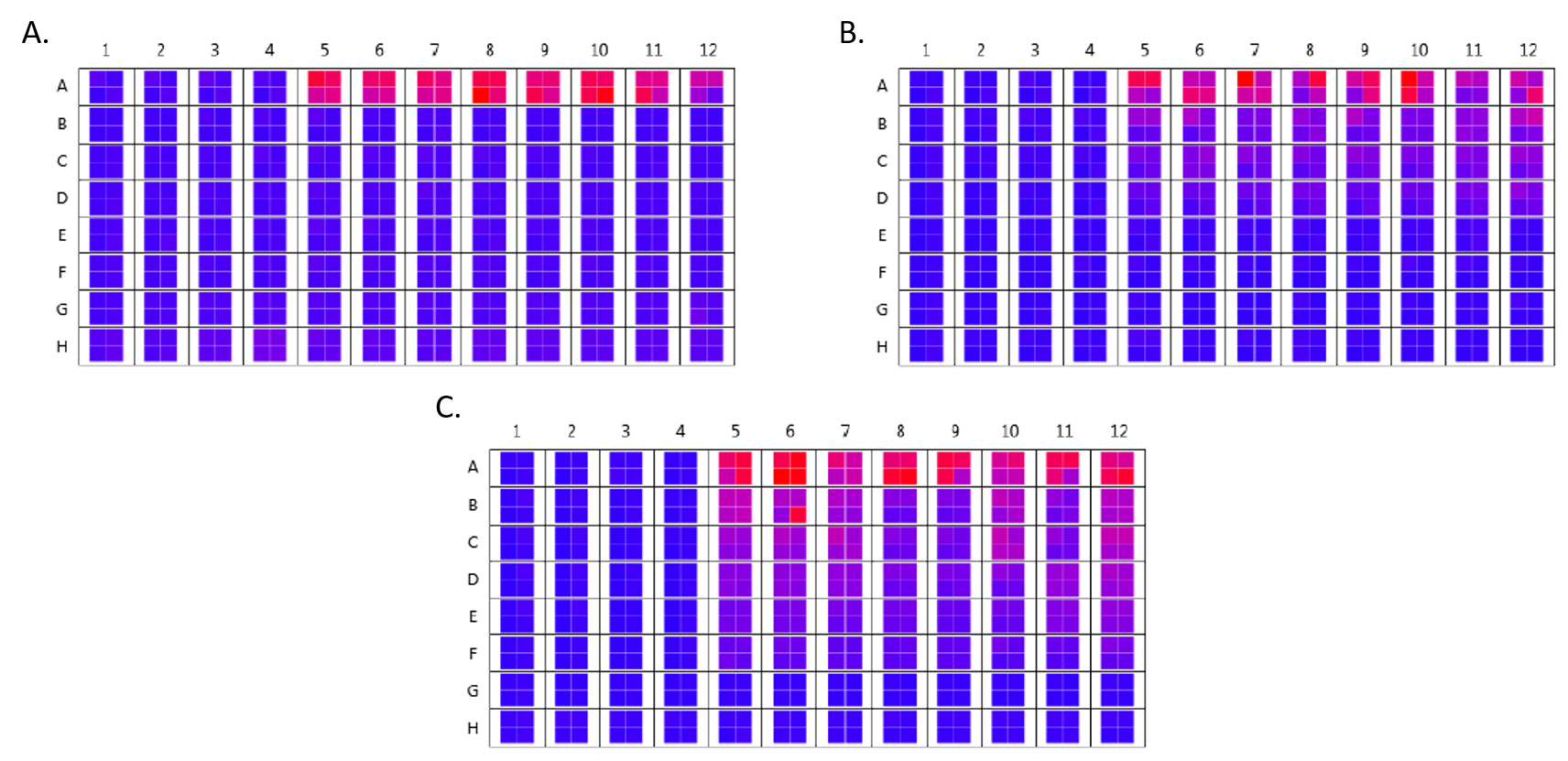

Supplement: Supplementary file 1 [file foods-11-00312-s001.zip › Figure S2.png]

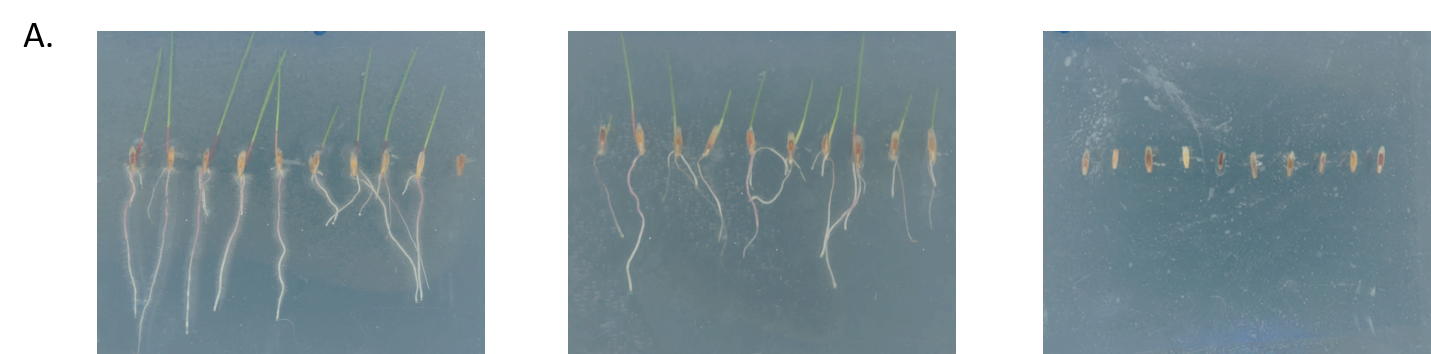

Supplement: Supplementary file 1 [file foods-11-00312-s001.zip › Figure S3A.png]

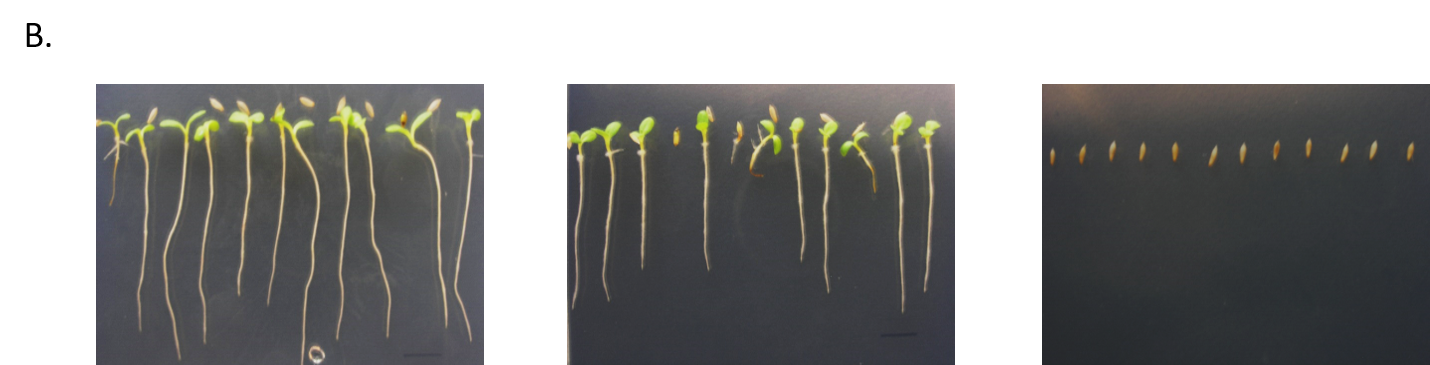

Supplement: Supplementary file 1 [file foods-11-00312-s001.zip › Figure S3B.png]
